# Combinatorial targeting of FGF and ErbB receptors blocks growth and metastatic spread of breast cancer models

**DOI:** 10.1186/bcr3379

**Published:** 2013-01-23

**Authors:** Amine Issa, Jason W Gill, Marinus R Heideman, Ozgur Sahin, Stefan Wiemann, Julien H Dey, Nancy E Hynes

**Affiliations:** 1 Mechanisms of Cancer, Friedrich Miescher Institute for Biomedical Research, Maulbeerstrasse 66, Basel 4058, Switzerland; 2 Division of Molecular Genome analysis, German Cancer Research Center, Im Neuenheimer Feld 580, Heidelberg 69120, Germany; 3 Current address: Department of Molecular and Cellular Oncology, The University of Texas MD Anderson Cancer Center, 1515 Holcombe Blvd, Houston TX 77030, USA; 4 University of Basel, Petersplatz 1, Basel 4003, Switzerland; 5 Current address: Nestlé Research Center, Vers-chez-les-Blanc, 1000 Lausanne 26, Switzerland

## Abstract

**Introduction:**

Targeting receptor tyrosine kinases (RTKs) with kinase inhibitors is a clinically validated anti-cancer approach. However, blocking one signaling pathway is often not sufficient to cause tumor regression and the effectiveness of individual inhibitors is often short-lived. As alterations in fibroblast growth factor receptor (FGFR) activity have been implicated in breast cancer, we examined in breast cancer models with autocrine FGFR activity the impact of targeting FGFRs in vivo with a selective kinase inhibitor in combination with an inhibitor of PI3K/mTOR or with a pan-ErbB inhibitor.

**Methods:**

Using 4T1 or 67NR models of basal-like breast cancer, tumor growth was measured in mice treated with an FGFR inhibitor (dovitinib/TKI258), a PI3K/mTOR inhibitor (NVP-BEZ235) or a pan-ErbB inhibitor (AEE788) individually or in combination. To uncover mechanisms underlying inhibitor action, signaling pathway activity was examined in tumor lysates and transcriptome analysis carried out to identify pathways upregulated by FGFR inhibition. Anti-phosphotyrosine receptor antibody arrays (P-Tyr RTK) were also used to screen 4T1 tumors.

**Results:**

The combination of dovitinib + NVP-BEZ235 causes tumor stasis and strong down-regulation of the FRS2/Erk and PI3K/Akt/mTOR signaling pathways. P-Tyr RTK arrays identified high levels of P-EGFR and P-ErbB2 in 4T1 tumors. Testing AEE788 in the tumor models revealed that the combination of dovitinib + AEE788 resulted in blockade of the PI3K/Akt/mTOR pathway, prolonged tumor stasis and in the 4T1 model, a significant decrease in lung metastasis. The results show that *in vivo *these breast cancer models become dependent upon co-activation of FGFR and ErbB receptors for PI3K pathway activity.

**Conclusions:**

The work presented here shows that in the breast cancer models examined, the combination of dovitinib + NVP-BEZ235 or dovitinib + AEE788 results in strong inhibition of tumor growth and a block in metastatic spread. Only these combinations strongly down-regulate the FGFR/FRS2/Erk and PI3K/Akt/mTOR signaling pathways. The resultant decrease in mitosis and increase in apoptosis was consistently stronger in the dovitinib + AEE788 treatment-group, suggesting that targeting ErbB receptors has broader downstream effects compared to targeting only PI3K/mTOR. Considering that sub-classes of human breast tumors co-express ErbB receptors and FGFRs, these results have implications for targeted therapy.

## Introduction

Members of the receptor tyrosine kinase (RTK) superfamily are often aberrantly expressed and/or activated in human tumors and many have been successfully targeted using antibody-based therapies or tyrosine kinase inhibitors (TKI) [1]. In breast cancer, ErbB2 has proven to be an excellent target; however, only 25% of cancer patients are eligible for an ErbB2-directed therapy [2,3]. Currently much effort is going into uncovering other RTKs that when inhibited could impact disease. The fibroblast growth factor receptors (FGFRs) and their ligands have been implicated in many different types of tumor, including breast cancer. Indeed, amplification of *FGFR1 *or *FGF3 *has been detected in approximately 10% or 15% of primary tumors respectively, while patients with *FRFR1 *amplification are more likely to develop distant metastasis [4], as such FGFRs are considered to be highly relevant therapeutic targets [5,6].

The 4T1 and 67NR mammary cancer cell lines are widely studied models for basal-like breast cancer that have similar genetic backgrounds but different metastatic potential. When implanted in Balb/c mice the 67NR cells form mammary tumors that do not metastasize, while the 4T1 mammary tumors are able to spread to and grow in distant organs [7]. We have previously shown that both tumor cell lines display autocrine FGFR activity due to co-expression of FGFRs and ligands. Using the FGFR selective inhibitor, dovitinib (TKI258) [8], we showed that the 4T1 and 67NR cancer cell lines are dependent upon FGFR signaling for proliferation and survival, and that mammary tumor outgrowth is significantly slower in dovitinib-treated mice [9]. While tumors from dovitinib-treated animals displayed a strong reduction in FRS2/Erk pathway signaling, the phosphatidyl inositol 3'kinase (PI3K)/Akt pathway showed little or no downregulation [9]. In the results presented here we further explored the role of the PI3K/Akt/mammalian target of rapamycin (mTOR) pathway and RTKs that regulate this pathway in the 4T1 and 67NR models.

We show that the combination of dovitinib with the PI3K/mTOR inhibitor, NVP-BEZ235 [10], strongly downregulates the FRS2/extracellular signal-regulated kinase (Erk) and PI3K/Akt/mTOR signaling pathways, resulting in high levels of apoptosis and tumor stasis. Using an unbiased approach to screen for active receptors, anti-phosphotyrosine receptor antibody arrays (P-Tyr RTK), we identified high levels of P-epidermal growth factor receptor (P-EGFR) and P-ErbB2 in the tumors. Testing the pan-ErbB inhibitor AEE788 [11] in the 4T1 and 67NR models revealed that only the combination of AEE788 and dovitinib resulted in blockade of the FRS2/Erk and PI3K/Akt/mTOR pathways, high levels of apoptosis with prolonged tumor stasis, and in the 4T1 model a highly significant decrease in lung metastasis. Our results suggest that *in vivo*, but not *ex vivo*, both breast cancer models become dependent upon co-activation of FGFR and ErbB receptors for PI3K/Akt/mTOR pathway activity, demonstrating the importance of the tumor environment in influencing receptor activity and response to targeted inhibitors. In the models we studied, optimal blockade of tumor growth and metastatic spread was only achieved by combining an FGFR inhibitor with the PI3K/mTOR inhibitor or with the pan-ErbB inhibitor. Considering that breast tumors co-express multiple RTKs including ErbB and FGFRs [12,13], these results have important implications for targeted therapy.

## Materials and methods

### Kinase inhibitors

The inhibitors dovitinib [8], NVP-BEZ235 [10] and AEE788 [11] were provided by Drs. D Graus-Porta, S-M Maira and G Caravatti (Novartis Institutes for Biomedical Research, Basel, Switzerland). All inhibitors were prepared as 10 mmol/L dimethyl sulfoxide (DMSO) stocks for *in vitro *use or diluted in the corresponding carrier for *in vivo *experiments.

### Cell lines, *in vivo *treatments and analysis

The 4T1 and 67NR cell lines [7] were maintained as described [9]. We examined the 4T1 cell line for mutations in *PI3KA*, *K-Ras *and *FGFR3*. We sequenced exons 9 and 20 of *PI3KCA*, exons 1 and 2 of *K-Ras *and exons 7, 10 and 15 of *FGFR3*; none of these exons were mutated. Animal experiments were performed according to the Swiss guideline governing animal experimentation and approved by the Swiss veterinary authorities. The 4T1 and 67NR cells (5 × 10^5 ^cells) were injected into the fourth mammary fat pad of 10-week-old BALB/c mice (Harlan Laboratories, Netherlands). Once palpable, tumors were measured daily and volume was calculated using the following formula: Volume = Height × ((Diameter/2)^2 ^× *π*)

Mice were randomly distributed into groups when tumors reached 50 to 100 mm^3^. Different groups were treated for the indicated times with different doses depending upon the experiment: vehicle (water or polyethylene glycol 300), dovitinib (per oral (p.o.), once daily) AEE788 (p.o., thrice weekly), NVP-BEZ235 (p.o., once daily), the combination of dovitinib (20 mg/kg) and AEE788 (40 mg/kg), or dovitinib (20 mg/kg) and NVP-BEZ235 (10 mg/kg). For experimental metastasis, 2.0 to 2.5 × 10^5 ^4T1 cells were injected into tail veins; 24 hrs later, mice were treated with PEG300 or NVP-BEZ235 (10 days at 20 mg/kg); alternatively, 7 days after injection, treatment was started for 11 days; dovitinib (20 mg/kg), NVP-BEZ235 (10 mg/kg), AEE788 (40 mg/kg), dovitinib/AEE788 or dovitinib/NVP-BEZ235. At the end, lungs were isolated and placed in Bouin's solution to visualize and count metastases (Leica MacroFluo Z6, Leica Microsystems, Heerbrugg, Switzerland). Results are reported as average number of nodules per group.

### Tumor serial transfer

Inhibitor-treated mice were sacrificed and tumors were digested for 1 hr at 37°C in Collagenase (1 mg/ml), Dispase (1 mg/ml) and DNAse (50 KU/ml) to a single cell suspension. Hematopoietic cells labeled with CD45-biotin (Biolegend, San Diego, USA) were removed from samples using anti-biotin magnetic bead depletion (EasySep, StemCell Technologies, Grenoble, France) and tumor cells were enriched via discontinuous percoll density gradient separation (GE Healthcare, Glattbrugg, Switzerland). Equal numbers of tumor cells were injected into recipient Balb/c mice. Tumors were visible by 7 days; tumor-take was 100%.

### Analysis of drug effect on circulating tumor cells

Circulating tumor cells in 4T1 tumor-bearing mice were quantified as described in [14] and collected cells were cultured in media supplemented with 60 μM 6-thioguanine to select for 4T1 cells [7]. After 14 days colonies were stained and counted.

### Immunofluorescence and image measurements

For immunohistological analysis, tumors were dissected and frozen in optimum cutting temperature compound (OCT) on a 2-methylbutane, dry ice bath. Cryosections (10 μm) were fixed in 1:1 methanol/acetone, blocked with 1% rat, donkey and goat serum and stained using antibodies for CD31-FITC (Biolegend, San Diego, USA. Clone 390), phosphorylated histone-H3-Alexa-Fluor647 (Biolegend, Clone HTA28), and cleaved caspase-3 (Cell Signaling Technology, Inc. Danvers, MA, USA 9661) with Alexa-Fluor594 conjugated anti-Rabbit IgG (Invitrogen, Lucerne, Switzerland) secondary. Sections were mounted with Prolong gold containing 4',6-diamidino-2-phenylindole (DAPI) (Invitrogen) and images acquired using an Axio Imager Z2 LSM700 confocal microscope (Zeiss, Feldbach, Switzerland). Images were analyzed using Image J (NIH, MD, USA). Image measurements were taken in 15 to 20 digital images (10 × objective lens) from three to four separate tumor specimens, with four images taken in each quadrant of the tumor perimeter and one in the center region. The area of CD31 or cleaved caspase-3 immunoreactivity was measured as the number of pixels above the fluorescence threshold (typically 15 to 30) as a proportion of total pixels within defined tumor boundaries; values were not influenced by tumor size. Phosphorylated histone-3-positive cells were manually counted, and the final values presented as an average number of cells per field.

### Gene expression analysis and pathway enrichment

The 4T1 tumors were taken from mice treated with vehicle, or with 15 or 40 mg/kg dovitinib for 2 and 8 hrs or for 1, 3 and 10 days. Total RNA was isolated from three tumors/treatment time points using RNeasy Mini Kits (Qiagen, Hilden, Germany) following the manufacturer's instructions. Sentrix MouseWG-6 V2 arrays (Illumina, San Diego, CA, USA) were used for expression profiling. Quality control of the RNA (Agilent Bioanalyzer), as well as labeling and array hybridization was performed at the DKFZ microarray Core Facility. Data were normalized with the variance stabilization transformation algorithm (Bioconductor vsn package) and genes with significant change (*P *< 0.05, log-fold-change > 0.5) were recorded. The microarray data have been submitted to Array-Express [E-MTAB-1260: EBI]. The Bioconductor limma package was used to identify differentially expressed genes and two-step regression (Bioconductor maSigPro package) was applied to identify genes with temporal expression changes. STRING [15] and DAVID Bionformatics Resources 6.7 [16] were used to map protein interactions and for functional gene enrichment, respectively. R-script was used to generate the plots for EGFR and its ligands (see Figure S5 in Additional file 1).

### Western blot analysis and RTK phosphorylation detection

Protein lysates were prepared and western analyses were performed as described [9]. The following antibodies were used: P-mTOR, P-ErbB2, P-FRS2, P-Akt, PERK1/2, P-S6, Akt, ERK1/2, S6 all from Cell Signaling, FRS2 (Santa Cruz Biotechnology, Inc. Dallas, Texas, USA) and ErbB2 [17]. Blots were probed using an appropriate horseradish peroxidase-conjugated secondary antibody (Amersham, GE Healthcare, Glattbrugg, Switzerland) and developed using Western Pico ECL substrate kit (GE Healthcare). Detection of phosphorylated RTKs in tumor lysates was performed using a Proteome Profiler Array kit (R&D systems, Abingdon, UK. cat. No. ARY014) as per the manufacturers protocol. Quantification of signal was determined using Image J software.

### Statistics

For determining statistical significance in all quantifications, non-parametric Mann-Whitney *U*-tests were used; all data are presented as mean ± SD. Data were considered significant for *P-*values < 0.05 and are denoted as follows: *P < 0.05, **P < 0.01.

## Results and discussion

### Effects of NVP-BEZ258 on the 4T1 and 67NR tumor models

Balb/c mice develop mammary tumors following injection of 4T1 and 67NR tumor cell lines into the mammary fat pad [7]. Both cell lines display autocrine FGFR signaling activity due to co-expression of ligands and receptors [9]. Treatment of 4T1 and 67NR tumor-bearing mice with the FGFR inhibitor dovitinib causes a significant reduction in tumor volume; however, tumor stasis was not observed (see Figure S1 in Additional file 1) as previously shown [9]. Since there was no detectable effect on P-Akt levels in dovitinib-treated tumors (see Figure S2 in Additional file 1), we tested the effects of NVP-BEZ235, a PI3K/mTOR inhibitor [10], in combination with dovitinib in these models.

**Figure 1:**
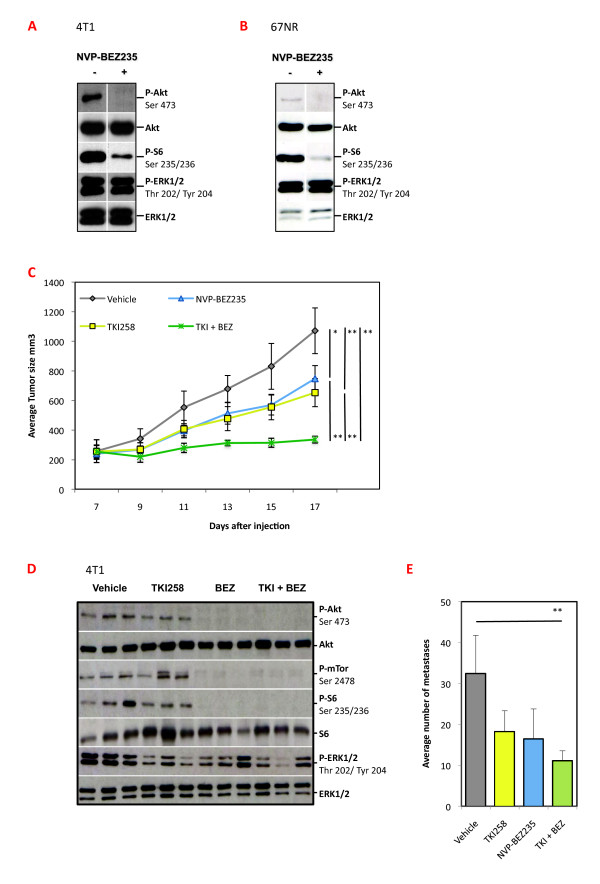
**Effect of fibroblast growth factor receptor (FGFR) and phosphatidyl inositol 3'kinase (PI3K)/mammalian target of rapamycin (mTOR) inhibitors on signaling and tumor outgrowth**. T1 (**A**) or 67NR (**B**) cell cultures were treated for 1 hr with 0.5 μM NVP-BEZ235 or control dimethyl sulfoxide (DMSO)-containing medium, lysates were prepared and a western analysis for the indicated proteins and phospho-proteins was performed. (**C) **Starting 7 days after injection of 4T1 cells in mammary fat pads, groups of tumor-bearing mice (*n *= 6) were treated for 14 days with vehicle (PEG300), dovitinib (TKI, 20 mg/kg), NVP-BEZ235 (10 mg/kg) or a combination of both, and tumor volume was determined; representative of two different experiments. (**D**) 4T1 tumor-bearing mice were treated with a single dose of the vehicle (PEG300), dovitinib (TKI, 20 mg/kg), NVP-BEZ235 (10 mg/kg) or a combination of both (TKI + BEZ) and sacrificed 2 hrs later. Tumor lysates were prepared from three independent mice per group and a western analysis for the indicated proteins and phospho-proteins (P) was performed. (**E**) Quantification of metastatic foci number in the lung tissue taken from animals at the end of the experiment in panel C. *N *= 6, representative of two separate experiments. *P < 0.05 **P < 0.01 (Mann-Whitney *U*-test).

Initially we tested *in vitro *effects of NVP-BEZ235 by treating 4T1 and 67NR cultures and examining signal pathway activity by western analyses. The Erk and PI3K/Akt/mTOR pathways are constitutively active in these cell lines [9]. Lysates from NVP-BEZ235-treated cultures showed significantly decreased levels of P-Akt and P-S6, readouts for PI3K pathway activity, whereas P-Erk, which is controlled by FGFR [9], is not affected (Figure 1A-B). The *in vivo *effects of the inhibitors on tumor outgrowth were also examined.

We have previously shown that dovitinib has dose-dependent anti-tumor activity as a single agent [9]. We initially tested different doses of NVP-BEZ235; however, doses higher than 10 mg/kg resulted in significant weight loss (not shown). Thus, in long-term experiments, NVP-BEZ235 and dovitinib were dosed at 10 mg/kg and 20 mg/kg, respectively. Groups of 4T1 tumor-bearing mice were treated daily for 14 days with individual inhibitors and with their combination. Tumor outgrowth was significantly slower in mice treated with individual inhibitors, but with combination treatment tumor stasis was observed (Figure 1C); importantly there were no significant changes in body weight (not shown). To examine pathway activity in the tumors, lysates were analyzed from three individual tumors taken from vehicle-control and inhibitor-treated mice. For this, a single dose of vehicle, dovitinib, NVP-BEZ235 or the dovitinib + NVP-BEZ235 combination was administered and tumors were collected 2 hrs later. Control tumors had high levels of P-Akt, P-mTor, P-S6 and P-Erk. Dovitinib-treated tumors had decreased P-Erk levels; as mentioned above this inhibitor has little or no impact on P-Akt levels. Treatment with NVP-BEZ235 alone or combined with dovitinib caused strong reduction in P-Akt, P-mTor, P-S6 and P-Erk (Figure 1D).

The effect of individual and combination treatment on metastasis was also analyzed by quantifying tumor nodules on lungs taken at the end of the 14-day treatment. Lungs from all the treatment groups had decreased numbers of metastases. However, in keeping with the strong effect of the combination on primary tumor outgrowth, only in this group was the decrease significant. In conclusion, the combination of dovitinib with NVP-BEZ235 caused tumor stasis and blocked signaling pathway activity in the tumors, as well as leading to a significant decrease in lung metastases.

### The combination of dovitinib and NVP-BEZ235 strongly blocks 4T1 tumor cell survival and intravasation

**Figure 2:**
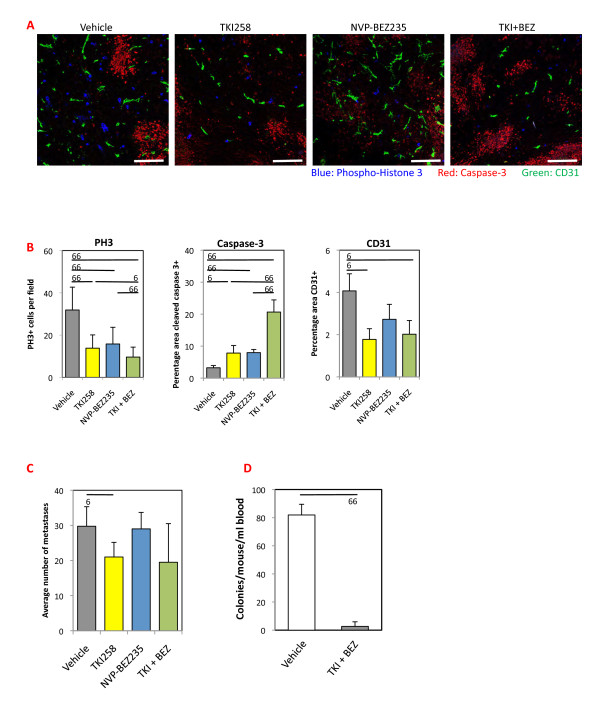
**Analyses of 4T1 tumors and lung metastases in mice treated with dovitinib, NVP-BEZ235 or the combination**. (**A**) 4T1 tumors from mice treated for 14 days with the inhibitors described in Figure 1C were harvested, and frozen sections were stained for proliferation (phospho-histone H3; blue), apoptosis (cleaved caspase 3; red) and endothelial cells (CD31; green). Scale bars, 100 μm. (**B**) Phosphorylated histone-3 (PH3)-positive cells were manually counted and expressed as the average number of cells per field. Proportion of tumor area expressing cleaved Caspase-3 and CD31 immunoreactivity was quantified using ImageJ. *N *= 3 to 4 tumors for each group with 5 fields analyzed per tumor (15 to 20 images from each treatment). (**C**) 2.5 × 10^5 ^4T1 cells were injected into tail veins and indicated treatments were started 7 days later for a total of 11 days. Lungs were harvested and metastases were quantified. *N *= 5, representative of two separate experiments. **(D) **After 14 days treatment with the combination of dovitinib (TKI, 20 mg/kg) and NVP-BEZ235 (10 mg/kg) or with vehicle, 4T1-tumor bearing mice were sacrificed, and blood was collected and plated. The graph shows the number and SD of tumor cell colonies growing/mouse/ml of blood; *N *= 5 to 9 animals per group. *P < 0.05 **P < 0.01 (Mann-Whitney *U*-test).

To uncover mechanisms underlying inhibitor activity, 4T1 tumors collected at the end of treatment (Figure 1C) were examined for proliferation, apoptosis and vessel density using P-Histone H3, cleaved Caspase-3 and CD31 respectively (Figure 2A). Quantification of stained sections revealed a significant decrease in proliferation and an increase in cell death in all treatment groups (Figure 2B), with the combination having the strongest impact on apoptosis (Figure 2B, middle panel). Tumor vascular area (CD31) was significantly reduced in dovitinib- and dovitinib + NVP-BEZ235-treated mice (Figure 2B, right panel). Since this inhibitor blocks not only FGFRs but also vascular endothelial growth factor receptors (VEGFRs) [8], the strong decrease in CD31 staining might reflect inhibition of both receptors. In these tumors, blood vessels showed fewer sprouts and were smaller, smoother and more uniform in size (Figure 2A, green). Taken together, these results suggest that the strong anti-tumor activity of combination treatment reflects high levels of tumor cell death.

Tumor-bearing mice treated with the dovitinib + NVP-BEZ235 showed significantly fewer lung metastases (Figure 1E). To directly analyze the impact of the inhibitors in the lungs, we analyzed experimental metastases. The 4T1 cells were injected into tail veins and 7 days later mice were treated with dovitinib or NVP-BEZ235, or the combination for 11 days, after which lung metastases were quantified. NVP-BEZ235- and control-treated mice had similar numbers of lesions; significantly fewer metastases were evident in dovitinib-treated mice, while for the combination treatment the *P*-value approached significance (*n *= 5, *P *< 0.06) (Figure 2C). Thus, once metastases are established in the lungs, only inhibition of FGFR signaling blocks tumor growth. We also examined intravasation by analyzing the number of circulating tumor cells in blood collected after 14 days of treatment. There were 40-fold fewer colonies growing from blood of dovitinib + NVP-BEZ235-treated mice compared with control (Figure 2D). Taken together, these results suggest that the strong impact of combination treatment on spontaneous lung metastasis (Figure 1E) is due to the strong effects on survival of cells in the primary tumor, combined with a block in their intravasation from the primary site and/or a lower survival potential of the circulating tumor cells.

### 4T1 and 67NR tumors retain prolonged sensitivity to the dovitinib + NVP-BEZ235 combination

**Figure 3:**
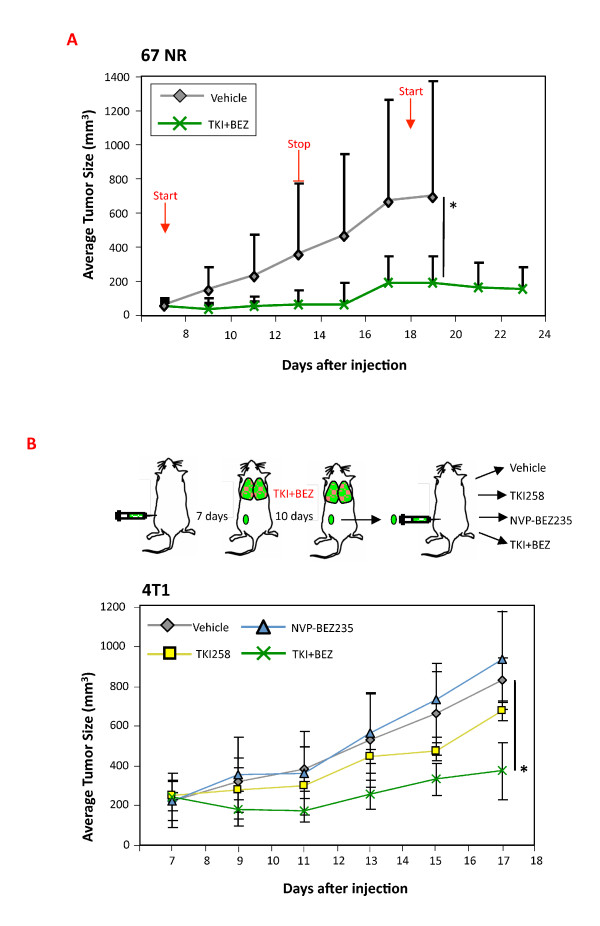
**4T1 and 67NR tumors retain prolonged sensitivity to treatment with the dovitinib + NVP-BEZ235 combination**. (**A**) Groups of 67NR tumor-bearing mice (*n *= 5) were treated daily with vehicle (PEG300), or with a combination of dovitinib (TKI, 20 mg/kg) and NVP-BEZ235 (10 mg/kg) for 7 days. Treatment was stopped for 5 days then resumed for 6 days and tumor growth was monitored. (**B**) Groups of 4T1 tumor-bearing mice (*n *= 6) were treated daily with vehicle (PEG300) or the combination of dovitinib (TKI, 20 mg/kg) + NVP-BEZ235 (10 mg/kg). After 10 days treatment, residual tumors were enzymatically digested, hematopoietic cell-depleted and 0.5 × 10^6 ^were re-injected into naïve mice (scheme in top panel). Starting 7 days after injection, groups of mice (*n *= 5) were treated daily for 11 days with (PEG300), dovitinib (TKI, 20 mg/kg), NVP-BEZ235 (10 mg/kg) or a combination of both, and tumor growth was monitored (lower portion of B). *P < 0.05 (Mann-Whitney *U*-test).

The 67NR tumor-bearing mice were also examined for their sensitivity to the dovitinib + NVP-BEZ235 combination. As observed in the 4T1 model, this treatment was very effective in blocking the PI3K/Akt/mTOR pathway in the tumors (see Figure S3 in Additional file 1) and after 7 days tumor growth was essentially blocked (Figure 3A). Mice removed from treatment were monitored and regrowth was observed after approximately 5 days. Importantly, the tumors responded well to a second treatment and even appeared to regress over the course of the last 4 days of the experiment (Figure 3A). Thus, 67NR tumors, like 4T1 tumors, are also very sensitive to treatment with the FGFR inhibitor in combination with the PI3K/mTOR inhibitor NVP-BEZ235.

We also examined 4T1 tumors for their sensitivity to the combination treatment after a treatment-free interval, using a different protocol. Following injection of 4T1 cells in the fat pad, lung metastases are already present at 7 days, when drug treatment is started, as determined by metastatic tumor growth following primary tumor resection (see Figure S4 in Additional file 1). Thus, to avoid complications associated with metastases, mice were killed and tumors removed after the first treatment period, and dissociated tumor cell suspensions were injected into naïve mice; once tumors were visible 7 days later, treatment was resumed (Figure 3B, top scheme). During the second treatment period (Figure 3B, lower panel), tumors in mice treated with dovitinib + NVP-BEZ235 initially appeared to regress; however, after approximately 7 days, regrowth was observed. In the same experiment, there was no response to individual inhibitor treatment (Figure 3B, lower panel). In summary, both 4T1 and 67NR models respond well to dovitinib + NVP-BEZ235 treatment. The 67NR model appears more sensitive than 4T1 tumors, since tumor stasis was observed over the time course.

### P-RTK analysis of the 4T1 cells and tumors

**Figure 4:**
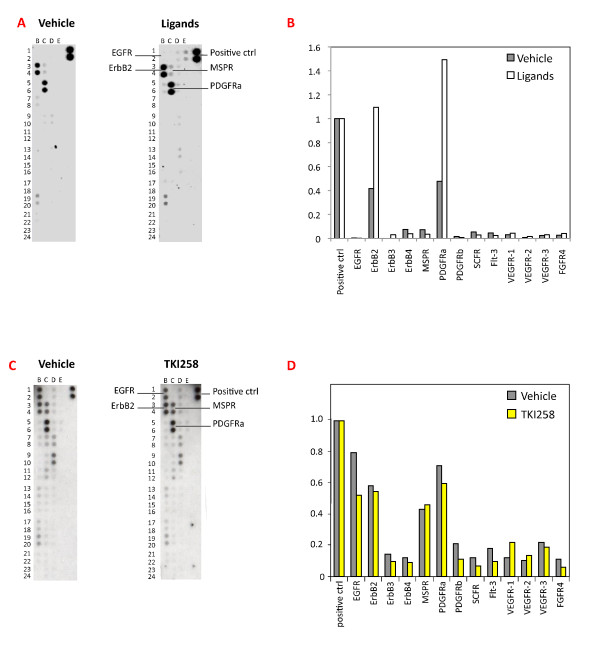
**Anti-phosphotyrosine receptor antibody (P-Tyr RTK) array analysis of 4T1 cells and tumors**. (**A**-**B**) P-RTK analysis on lysates of 4T1 cultures either growing in low serum (Vehicle) or stimulated for 15 minutes with 20 ng/ml EGF, 12 nM HRG or 20 ng/ml platelet-derived growth factor (PDGF) (Ligands). Lysates from individual ligand-treated cells were pooled prior to analysis. **(B) **Quantification of the signal intensity of the indicated p-RTKs is shown in comparison to positive control, set as 1. (**C-D**) Three control (Vehicle) and 50 mg/kg dovitinib-treated (TKI258) 4T1 tumors were harvested after 3 days treatment, and lysates were pooled and analyzed on P-RTK filters. (**D**) Quantification of the intensity of signal of indicated p-RTKs is shown, in comparison to positive control set as 1.

Blocking FGFR activity in combination with PI3K/mTOR inhibition was very effective in decreasing tumor growth. Our next goal was to uncover a tyrosine kinase receptor that when inhibited would block PI3K pathway activity. To approach this, we used anti-phosphotyrosine receptor antibody arrays (P-Tyr RTK) to screen for activity across a panel of RTKs in 4T1 cultured cells and tumors. In lysates from cell cultures, high basal levels of P-ErbB2 and P-platelet-derived growth factor receptor alpha (PDGFRa) were detected in vehicle control cells and their P-Tyr content increased in response to their activating ligands Heregulin (HRG), PDGF and EGF (Figure 4A, quantified in 4B); although P-EGFR (B1 to 2) was only visible on a longer exposure. The other RTKs, including FGFR2 (B9 to 10) and FGFR3 (B11 to 12), showed little or no P-Tyr. Using mass spectrometry and a phospho-proteomic screen, we have previously shown that FGFR-1, -2 and -3, which are expressed in the cells, each contain Tyr-P [9]; apparently their level is too low to detect with this RTK array. High levels of P-ErbB2 and P-PDGFRa were also detected in 4T1 tumor lysates (Figure 4C, vehicle). Interestingly, novel P-RTKs that were not detected in lysates from cell cultures, including P-EGFR, P-macrophage-stimulating protein receptor (P-MSPR), and to a lesser extent P-VEGFR3 (D7 to 8) and P-musk receptor (P-MuSK) (D9 to 10), were also found in the tumors (Figure 4C, vehicle, quantified in 4D). Tumors were analyzed 10 days after 4T1 injection so there would be sufficient time for ligands from the tumor environment [18] to influence their activity.

We also examined 10-day 4T1 tumors in mice that were treated the last three days with dovitinib, to see if blocking FGFR would impact on activation of other RTKs. No significant differences in the control compared to the inhibitor-treated tumors were observed (Figure 4C, TKI258, quantified in 4D). This result was unexpected, in particular for EGFR, since a transcriptome analysis revealed that EGFR, and the ligands amphigegulin (Areg), heparin binding EGF (HB-EGF) and TGFalpha were rapidly and significantly upregulated in dovitinib-treated tumors (see Figure S5 in Additional file 1). No significant alterations in RNA levels of any other RTK-ligand network were found in this analysis.

### 4T1 and 67NR tumors are sensitive to the combination of dovitinib and AEE788

**Figure 5:**
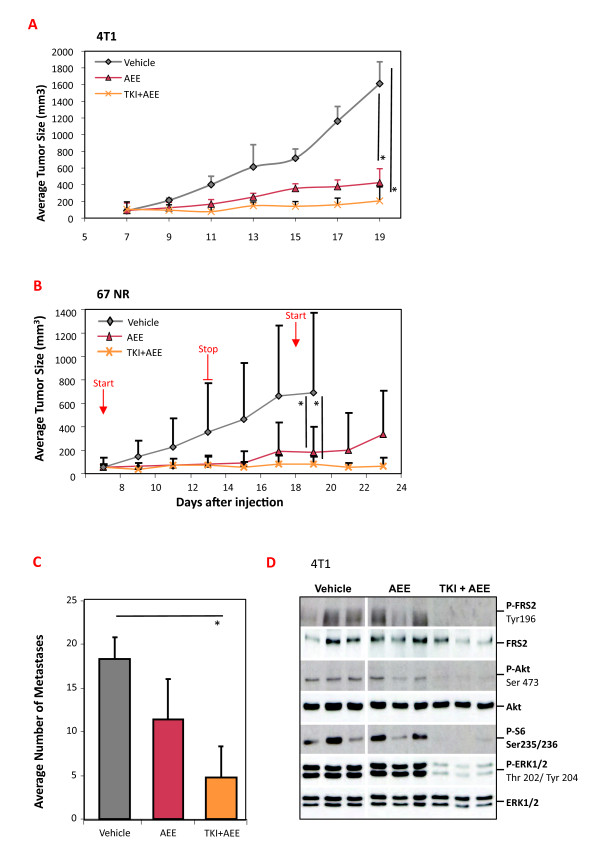
**Effect of dovitinib, AEE788 or the combination of both on 4T1 and 67NR tumors**. (**A) **Groups of 4T1 tumor-bearing mice (*n *= 6) were treated for 12 days with PEG300 (Vehicle), AEE788 (50 mg/kg) or a combination of dovitinib (TKI, 40 mg/kg) + AEE788. AEE788 was administered 3×/week; dovitinib daily, and tumor volume was determined; representative of two experiments. (**B) **Groups of 67NR tumor-bearing mice (*n *= 5) were treated with PEG300 (Vehicle), AEE788 (50 mg/kg) or a combination of dovitinib (TKI, 40 mg/kg) + AEE788. Treatment was performed through days 7 to 13 and days 18 to 23. Dovitinib and vehicle were dosed daily; AEE788 was administered on days 7, 9, 11 and 13, then on day 18, 20 and 22, and tumor volume was determined. (**C**) Quantification of the number of metastatic foci covering lungs of mice from the experiment in Panel A. *N *= 6, representative of two separate experiments. (**D) **4T1 tumor-bearing mice were treated with PEG300, a single dose of AEE788 (50 mg/kg) or a combination of dovitinib (TKI, 40 mg/kg) + AEE788, then sacrificed 2 hrs later. Tumor lysates were prepared from three mice per group and a western analysis for the indicated proteins and phospho-proteins (P) was performed. *P < 0.05 (Mann-Whitney *U*-test).

We decided to focus on ErbB receptors, since ErbB2 signals strongly to the PI3K pathway through ErbB3 [19], and pan-ErbB inhibitors are in clinical use [20]. For our work, we used AEE788, which has been shown to block EGFR and ErbB2 activity [11,21]. Preliminary testing with AEE788 revealed good anti-tumor activity in the 4T1 model, and a decrease in P-ErbB2 levels was readily detected in tumor lysates from AEE788-treated mice (see Figure S6 upper panel in Additional file 1). Groups of 4T1 and 67NR tumor-bearing mice were treated long-term with AEE788 or with the combination of dovitinib + AEE788. In both the 4T1 and the 67NR tumor models we observed significantly impaired tumor outgrowth with single agent AEE788 as well as with the combination, the latter treatment consistently showing stronger anti-tumor activity (Figure 5A and 5B). The 4T1 tumor-bearing mice treated with AEE788 alone had fewer lung metastases, but there was a stronger, significant effect in mice treated with the combination. We performed intermittent dosing in the 67NR model and observed tumor stasis over the course of three weeks in the combination treated group (Figure 5B). An analysis of signaling proteins in 4T1 tumors was also undertaken. Interestingly, there was no consistent decrease in P-Akt levels in tumors from AEE788 treated mice (Figure 5D) (see also Figure S6 lower panel in Additional file 1), which was surprising since *in vitro *treatment of 4T1 cells with AEE788 does block this pathway (see Figure S7 in Additional file 1). Only in the combination treated group did we observe a strong decrease in P-Akt and P-S6 (Figure 5D). As expected there was also a decrease in P-FRS2 and P-Erk levels in the combination-treated group, due to dovitinib treatment (Figure 5D). Taken together the results show that, concomitant inhibition of ErbB receptors and FGFRs has strong anti-tumor activity in both the 4T1 and 67NR models. Moreover, blocking ErbB RTK activity is not sufficient to lower PI3K pathway activity, only when combined with dovitinib was this achieved.

**Figure 6:**
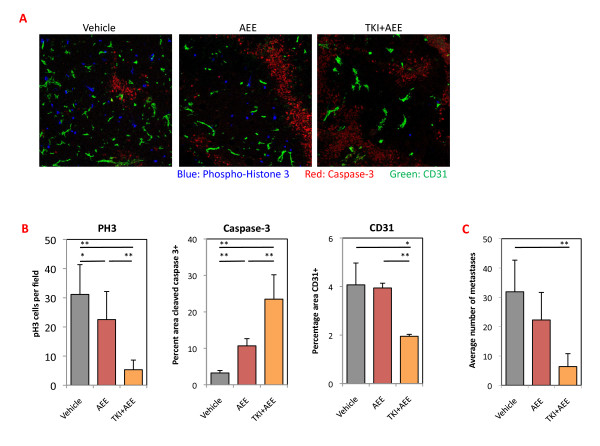
**Analysis of 4T1 tumors from mice treated with AEE788 + dovitinib**. (**A**) Frozen sections from 4T1 tumors harvested after 14 days of treatment as described in Figure 5A were stained for proliferation (phospho-histone H3; blue), apoptosis (cleaved caspase 3; red) and endothelial cells (CD31; green). Scale bars, 100 μm. (**B) **Phosphorylated histone-3 (PH3)-positive cells were manually counted and expressed as the average number of cells per field. Proportion of tumor area expressing cleaved Caspase-3 and CD31 immunoreactivity were quantified using ImageJ. *N *= 3 to 4 tumors for each group with five fields analyzed per tumor (15 to 20 images from each treatment). Bars represent mean ± SD in each treatment group. (**C**) 2.5 × 10^5 ^4T1 cells were injected into tail veins and indicated treatments were started 7 days later for a total of 11 days. Lungs were harvested and metastases were quantified. *N *= 5, representative of two separate experiments. *P < 0.05; **P < 0.01 (Mann-Whitney *U*-test).

The 4T1 tumors were collected at the endpoint and examined for proliferation, apoptosis and vessel density (Figure 6A). Quantification of P-Histone H3, cleaved Caspase-3 and CD31 revealed a significant decrease in cell proliferation and an increase in cell death, which was most prominent and significant in the dovitinib + AEE788-treated group (Figure 6B). The tumor vasculature area and morphology were significantly altered only after combination treatment, primarily as a result of the action of dovitinib as described earlier (Figure 2C). In the experimental metastasis model, treatment with AEE788 alone did not significantly lower the number of lung metastases, while the combination of dovitinib + AEE788 caused a highly significant decrease. These results support the hypothesis that combination therapy effectively inhibits primary tumor outgrowth by impairing proliferation and cell survival; while lung metastases are also very sensitive to blockade of both receptors.

## Conclusions

Targeting RTKs with antibodies or kinase inhibitors is a clinically validated anti-cancer approach; however, the effectiveness of individual inhibitors is often short-lived and resistance emerges. Experimental approaches have revealed numerous feedback loops in tumor cells and have shown that blocking one signaling pathway, be it the receptor [22,23] or downstream targets [24,25], is not sufficient to cause tumor regression, thereby allowing resistant cells to emerge. Moreover, inhibition of Akt or PI3K has been shown to increase the activity of multiple RTKs [24]. Taken together, it appears that the utility of single pathway inhibitors might be limited and that resistance to RTK inhibitors may often be due to activation of other RTKs that restore signaling [23]. Indeed, we and others have shown experimentally that ligand activation of EGFR or ErbB2/ErbB3 heterodimers overcomes the inhibitory effects of trastuzumab by stimulating downstream signaling pathways [26-28].

The 4T1 and 67NR models have been useful for examining the impact of FGFR inhibition on tumor growth and metastatic spread [9]. We previously showed that blocking FGFR *in vitro *was sufficient to inhibit Erk and PI3K signaling and to induce cell death via blockade of the latter pathway [9]. *In vivo *targeting of FGFR significantly slows tumor growth, but neither tumor stasis nor strong inhibition of PI3K/Akt signaling was observed [9]. As we show here, the PI3K/mTOR inhibitor NVP-BEZ235 robustly blocks this pathway and the combination of dovitinib + NVP-BEZ235 had significantly better anti-tumor and anti-metastatic activity than treatment with single inhibitors. It is becoming clear that *in vivo *responses to kinase inhibitors is optimal only when tumors show high levels of apoptosis [29]. Indeed, we show here that the most durable tumor responses and the highest levels of apoptosis were observed in mice treated with the FGFR inhibitor in combination with either the PI3K/mTOR inhibitor or the pan ErbB inhibitor. For both treatment modalities strong inhibition of the FGFR/FRS2/Erk pathway and the PI3K/Akt/mTOR pathway was observed.

An important goal of this work was to uncover a tyrosine kinase receptor that when inhibited would block PI3K/Akt/mTOR pathway activity. The ErbB RTKs were interesting to target for different reasons: EGFR and ErbB2 are both active in the tumors; ErbB2 signals strongly to the PI3K pathway through ErbB3 [19], and pan-ErbB inhibitors that block all three receptors are in clinical use [20]. Thus, we were surprised to find that the pan-ErbB inhibitor AEE788 [11,21] when given alone blocked ErbB receptor activity, but had no impact on PI3K/Akt signaling. Only the combination of AEE788 with dovitinib caused a significant block in the PI3K/Akt/mTOR pathway and resulted in strong anti-tumor activity. When AEE788 is dosed as a single agent it is possible that one of the other active RTKs maintains PI3K/Akt signaling. Indeed, the fact that addition of dovitinib to AEE788 did block the pathway, suggests that this is the case. We detected other RTKs with moderate levels of P-Tyr in 4T1 tumors and specific inhibitors for two of them, PDGFR and VEGFR, are available. We have already tested *in vivo *activity of the VEGFR inhibitor PTK787 [30], but as a single agent we found no change in 4T1 tumor outgrowth [9]. It will be interesting to examine the potential of blocking PDGFR or VEGFR in combination with AEE788 or dovitinib in future work.

It is interesting to compare the effects of the different inhibitors. First, the combination of dovitinib + AEE788 appears to be somewhat more effective than that of dovitinib + NVP-BEZ288. An analysis of the phosphorylation status of signaling proteins did not reveal major differences in tumors from these treatment groups; both combinations efficiently block the PI3K/Akt/mTOR pathway. However, the decrease in mitosis and increase in apoptosis was consistently stronger in the dovitinib + AEE788 treatment group, suggesting that targeting ErbB receptors has broader downstream effects compared to targeting only PI3K/mTOR. In experiments aimed at testing the durability of treatment response, this combination was also more effective. Indeed, 67NR tumors from mice treated with dovitinib + AEE788 remained static in the timeframe of our studies. Second, in tumors from dovitinib-treated mice, alone or with both combinations, we observed a significant decrease in CD31 staining, which was accompanied by changes in vessel morphology. NVP-BEZ235 has been shown to impact on tumor vessel permeability [31], but as a single agent in our studies, we did not observe significant changes in the vessels. Since dovitinib also targets VEGFRs [8,11] we have previously tested the effects of another more selective VEGFR inhibitor, PTK787 [32] and found that this inhibitor had no effect on 4T1 tumor outgrowth [9]. However, in the work we present here we consider it possible that the decreased CD31 staining in tumors from dovitinib-treated mice might be due to the combined effects of blocking VEGFR and FGFR activity. Third, looking at the effects of the inhibitors on 4T1 metastasis, a few conclusions can be made. After tail vein injection of 4T1 cells, the combination of dovitinib + AEE788 is clearly the best treatment of those tested, suggesting that in the lung environment tumor cells continue to be dependent on ErbB and FGF RTKs. In contrast, we were surprised to see that once 4T1 cells colonized the lungs, NVP-BEZ235 treatment had no effect on metastatic growth. In primary tumors, treatment with NVP-BEZ235 or dovitinib had similar significant effects on proliferation and apoptosis, albeit lower than the combination. These results suggest that in the lung environment the PI3K/mTOR pathway is not so important for tumor cell growth. Finally, the dovitinib + NVP-BEZ235 combination strongly inhibited intravasation from the primary tumor into the bloodstream and/or tumor cell survival, which very likely contributes to the low number of spontaneous lung metastases in these animals.

Multiple RTKs are often active in cancer cells and combinations of RTK inhibitors have been shown to be better at blocking PI3K/Akt/mTOR signaling than individual inhibitors (for example, by Stommel *et al. *[33]). Considering FGFR and ErbB receptors as targets, non-small cell lung cancer cell lines have been shown to respond better to combinations targeting both, compared to individual treatments [34]. Our previous work with human breast cancer cell lines showed that the combined inhibition of FGFR and ErbB receptors caused a complete loss of PI3K/Akt/mTOR pathway activity and a robust block in *in vitro *proliferation [35]. Using a panel of tumor-derived cell lines with defined sensitivity to ErbB kinase inhibitors, it was shown that many of these tumor cell lines are rescued by FGF addition [23]. These *in vitro *results clearly show that FGFR activation can, in many cases, circumvent ErbB receptor inhibition. Does this occur in cancer patients? In a small group of ErbB2-positive breast cancer patients treated with lapatinib, those whose tumors had elevated levels of FGFR2 had a shorter time to progression than the low FGFR2 group [36]. The amount of genome-wide information available for breast tumors is increasing at a rapid pace and should help in choosing patients for whom simultaneous inhibition of ErbB and FGF receptors might be appropriate. *FGFR *amplification has been found in some basal-like breast cancers, a group that also has *EGFR *amplification [12]. *FGFR1 *is preferentially amplified in estrogen receptor-positive tumors and in our experience these often co-express ErbB family members. Indeed, some breast tumors with copy number changes in both *ERBB2 *and *FGFR1 *were recently described [13]. In addition to genomic alterations including copy number changes or mutations, ligand-mediated receptor activation might also play an important role. It has been known for many years that FGF8 and FGF10, both ligands for FGFR2, are overexpressed in human breast tumors [37,38], suggesting that antibodies to screen for active FGFR2 would be very useful. There are many useful diagnostic tools for identifying ErbB receptor alterations in human tumors. In the future the development of additional reagents that can be used to predict FGFR activation in tumors would be an important area to pursue.

In the work presented here we show that combinatorial inhibition of FGFR and ErbB receptors has a very significant impact on the *in vivo *tumor growth and metastatic spread of breast cancer models. Considering the emerging evidence that breast tumors co-express ErbB and FGFRs, our results have important implications for targeted therapy. There are multiple ErbB family inhibitors available for clinical use, and additional, more selective FGFR inhibitors, such as NYP-BGJ398, are now starting clinical development [39]. In the future it should be possible to select breast cancer patients for whom combination therapy would be appropriate.

## Abbreviations

Areg: amphigegulin; DAPI: 4',6-diamidino-2-phenylindole; DMSO: dimethyl sulfoxide; EGFR: epidermal growth factor receptor; Erk: extracellular signal-regulated kinase; FGFR: fibroblast growth factor receptor; HB-EGF: heparin-binding EGF; HRG: Heregulin; MSPR: macrophage-stimulating protein receptor; mTOR: mammalian target of rapamycin; MuSK: musk receptor; OCT: optimum cutting temperature compound; PDGF: platelet-derived growth factor; PI3K: phosphatidyl inositol 3'kinase; p.o.: per oral; P-Tyr RTK: anti-phosphotyrosine receptor antibody arrays; RTK: receptor tyrosine kinase; TGFalpha: transforming growth factor alpha; TKI: tyrosine kinase inhibitor; VEGFR: vascular endothelial growth factor receptor.

## Competing interests

The authors declare that they have no competing interests.

## Authors' contributions

AI and JG designed and carried out the *in vivo *work and the analyses of the tumors and metastases described in this manuscript. JD helped with some of the initial *in vivo *experiments and discussions. AI performed western analyses and PCR analyses and phosphor-RTK analyses on tumors. RH performed the phospho RTK analysis on cell lines. SO and SW performed the microarray studies. NH conceived the study, participated in the experimental design and helped to draft the manuscript. All authors have read and approved the manuscript.

## Supplementary Material

Additional file 1 **Figure **1**Groups of 4T1 tumor-bearing mice (*n *= 3) were treated for 7 days with vehicle or dovitinib (TKI, 50 mg/kg) and tumor size was monitored**. P < 0.05 (Mann-Whitney *U*-test). The results are similar to what has been previously published [9]. **Figure **2**4T1 tumor-bearing mice were treated with a single dose of dovitinib (TKI, 40 mg/kg) or with the vehicle for 2 hours**. Tumor lysates were prepared from three mice for each group and a western analysis for the indicated proteins and phospho-proteins (P) was performed. **Figure **3**67NR tumor-bearing mice were treated once with vehicle control or the combination of dovitinib (TKI, 20 mg/kg), + NVP-BEZ235 (10 mg/kg) for 2 hours**. Tumor lysates were prepared from two mice for each group and a western analysis for the indicated proteins and phospho-proteins (P) was performed. **Figure **4**Seven days after injection of 4T1 cells into mammary fat pads of 10 Balb/c females, tumors from 5 mice were resected; the other five mice served as control**. After 10 days all mice were killed, lungs were harvested and the metastatic foci number in the lung tissue was quantified. **Figure **5**Transcriptome analysis on 4T1 tumor-bearing mice treated for the indicated times with dovitinib at a low (15 mg/kg) and high dose (40 mg/kg)**. The Bioconductor limma package was used to identify differentially expressed genes and two-step regression (Bioconductor maSigPro package) was applied to identify genes with temporal expression changes. DAVID Bionformatics Resources 6.7 [16] was used for functional gene enrichment. R-script was used to generate the plots for epidermal growth factor receptor (EGFR) and its ligands. **Figure **6**Groups of 4T1 tumor-bearing mice were treated with AEE788 (50 mg/kg), dovitinib (TKI, 40 mg/kg) or vehicle control and tumors were harvested 2 hours later**. A western analysis of the indicated proteins and phospho-proteins (P) was carried out. **Figure 7 67NR cell cultures were left untreated (-) or pretreated for one hour with 1 μM AEE788 (+), then treated or not with HRG (100 nM for 10 minutes)**. Lysates were prepared and analyzed by western blot for the indicated proteins and phospho-proteins (P). Click here for file
