# Supplementary material for: Combinatorial targeting of FGF and ErbB receptors blocks growth and metastatic spread of breast cancer models
Source: Breast Cancer Res. 2013 Jan 23;15(1):R8. doi: 10.1186/bcr3379 (PMC3672810; doi:10.1186/bcr3379)
Supplement: Additional file 1 — Figure 1Groups of 4T1 tumor-bearing mice (n = 3) were treated for 7 days with vehicle or dovitinib (TKI, 50 mg/kg) and tumor size was monitored. P < 0.05 (Mann-Whitney U-test). The results are similar to what has been previously published [9]. Figure 24T1 tumor-bearing mice were treated with a single dose of dovitinib (TKI, 40 mg/kg) or with the vehicle for 2 hours. Tumor lysates were prepared from three mice for each group and a western analysis for the indicated proteins and phospho-proteins (P) was performed. Figure 367NR tumor-bearing mice were treated once with vehicle control or the combination of dovitinib (TKI, 20 mg/kg), + NVP-BEZ235 (10 mg/kg) for 2 hours. Tumor lysates were prepared from two mice for each group and a western analysis for the indicated proteins and phospho-proteins (P) was performed. Figure 4Seven days after injection of 4T1 cells into mammary fat pads of 10 Balb/c females, tumors from 5 mice were resected; the other five mice served as control. After 10 days all mice were killed, lungs were harvested and the metastatic foci number in the lung tissue was quantified. Figure 5Transcriptome analysis on 4T1 tumor-bearing mice treated for the indicated times with dovitinib at a low (15 mg/kg) and high dose (40 mg/kg). The Bioconductor limma package was used to identify differentially expressed genes and two-step regression (Bioconductor maSigPro package) was applied to identify genes with temporal expression changes. DAVID Bionformatics Resources 6.7 [16] was used for functional gene enrichment. R-script was used to generate the plots for epidermal growth factor receptor (EGFR) and its ligands. Figure 6Groups of 4T1 tumor-bearing mice were treated with AEE788 (50 mg/kg), dovitinib (TKI, 40 mg/kg) or vehicle control and tumors were harvested 2 hours later. A western analysis of the indicated proteins and phospho-proteins (P) was carried out. Figure 7 67NR cell cultures were left untreated (-) or pretreated for one hour with 1 μM AEE788 (+), then [file bcr3379-S1.PDF]

**Issa et.al.**
**Combinatorial targeting of FGF and ErbB receptors blocks growth and metastatic spread of breast cancer models**

# Figure S1

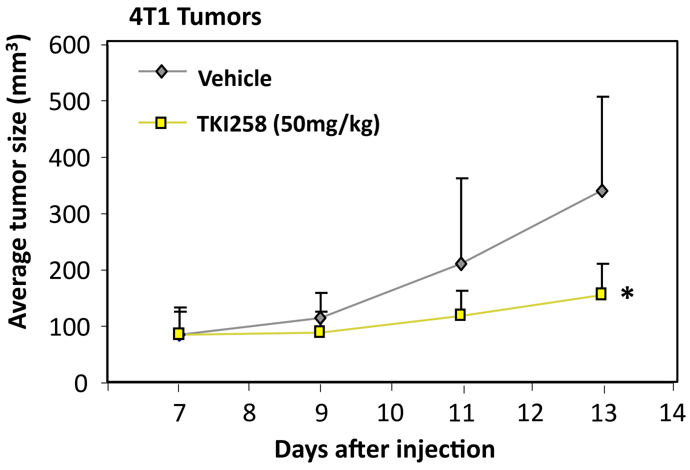


**Additional file 1, Figure S1**- Groups of 4T1 tumor-bearing mice (N=3) were treated for 7 days with vehicle or TKI258 (50 mg/kg) and tumor size was monitored. P<0.05 (Mann-Whitney U test). The results are similar to what has been previously published [9].

# Figure S2

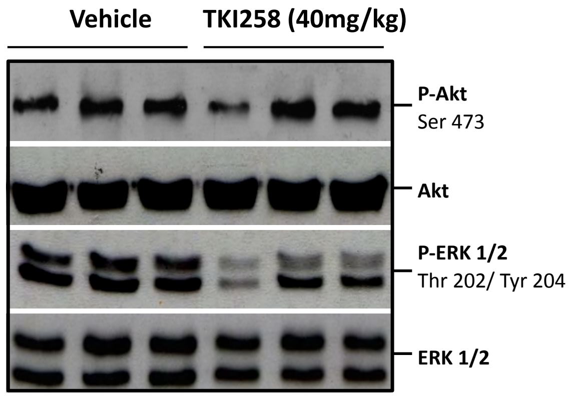


**Additional file 1, Figure S2**- 4T1 tumor bearing mice were treated with a single dose of TKI258 (40 mg/kg) or with the vehicle for 2 hours. Tumor lysates were prepared from 3 mice for each group and a western analysis for the indicated proteins and phospho-proteins (P) was performed.

# Figure S3

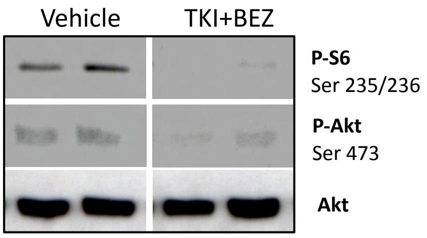


**Additional file 1, Figure S3**- 67NR tumor bearing mice were treated once with vehicle control or the combination of TKI258 (20 mg/kg), + NVP-BEZ235 (10 mg/kg) for 2 hours. Tumor lysates were prepared from 2 mice for each group and a western analysis for the indicated proteins and phospho-proteins (P) was performed.

# Figure S4

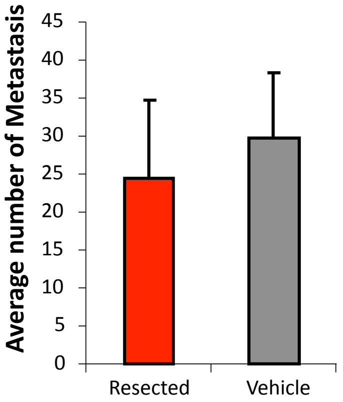


**Additional file 1, Figure S4 –**Seven days after injection of 4T1 cells into mammary fat pads of 10 Balb/c females, tumors from 5 mice were resected; the other 5 mice served as control. After 10 days all mice were killed, lungs were harvested and the metastatic foci number in the lung tissue was quantified.

# Figure S5

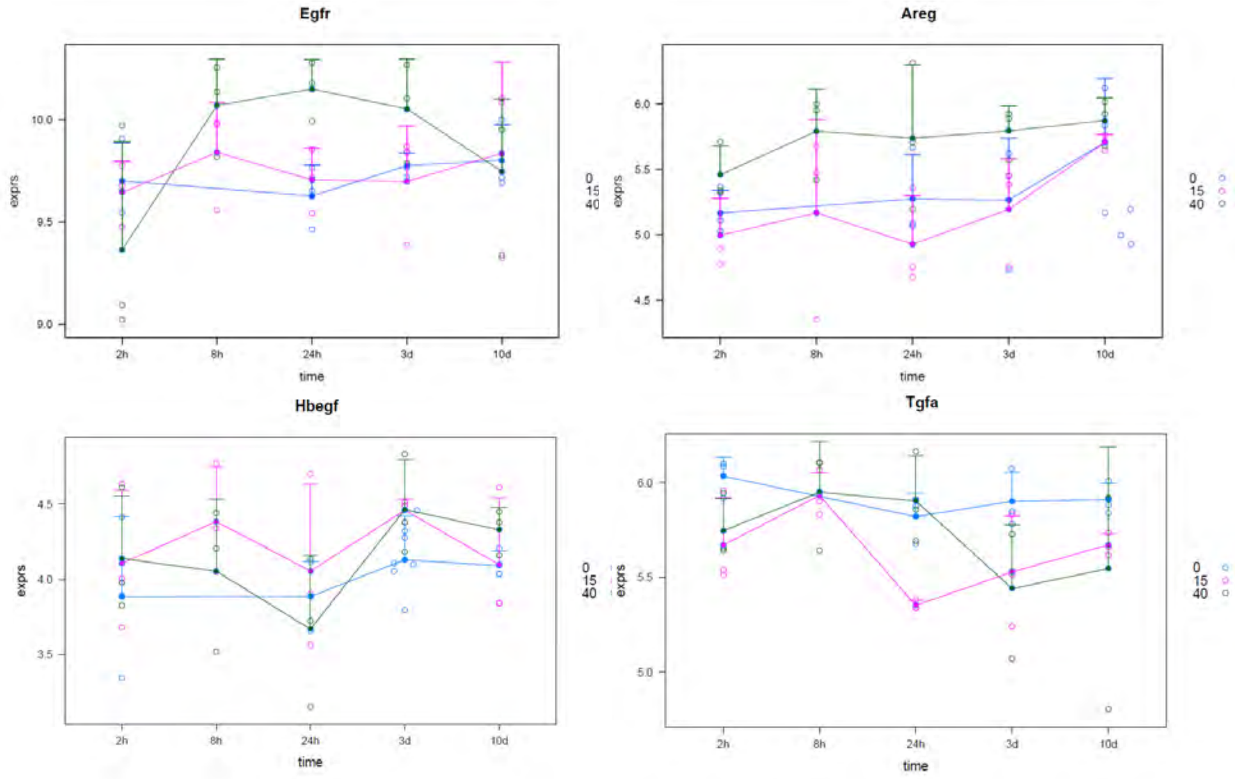


**Additional file 1, Figure S5 –** Transcriptome analysis on 4T1 tumor bearing mice treated for the indicated times with TKI258 at a low (15mg/kg) and high dose (40mg/kg). The Bioconductor limma package was used to identify differentially expressed genes and a two-step regression (Bioconductor maSigPro package) was applied to identify genes with temporal expression changes. DAVID Bionformatics Resources 6.7 [16] was used for functional gene enrichment. R-script was used to generate the plots for EGFR and its ligands

# Figure S6

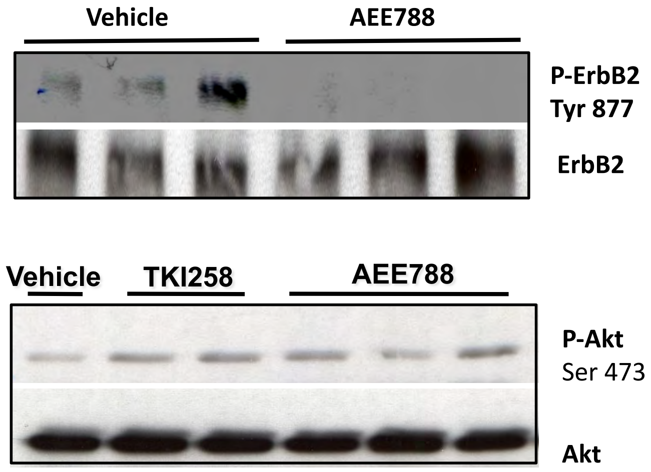


**Additional file 1, Figure S6-** Groups of 4T1 tumor bearing mice were treated with AEE788 (50mg/kg), TKI258 (40mg/kg) or vehicle control and tumors were harvested 2 hrs later. A western analysis of the indicated proteins and phospho-proteins (P) was carried out.

# Figure S7

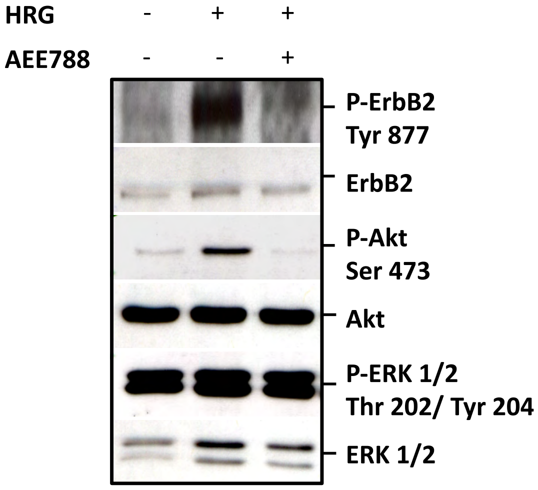


**Additional file 1, Figure S7-** 67NR cell cultures were left untreated (-) or pretreated 1 hour with 1µM AEE788 (+), then treated or not with HRG (100 nM for 10 min). Lysates were prepared and analyzed by a western for in the indicated proteins and phospho-proteins (P).
